# Successful Bailout of Catheter-Induced Dissection in Acute Myocardial Infarction Resulting From a Nondominant Right Coronary Artery Occlusion

**DOI:** 10.1155/cric/1091601

**Published:** 2024-12-16

**Authors:** Hiroshi Abe, Tadao Aikawa, Ken Yokoyama, Tohru Minamino

**Affiliations:** ^1^Department of Cardiology, Juntendo University Urayasu Hospital, Urayasu, Japan; ^2^Department of Cardiovascular Biology and Medicine, Juntendo University Graduate School of Medicine, Tokyo, Japan

**Keywords:** acute myocardial infarction, catheter-induced dissection, nondominant right coronary artery

## Abstract

A 48-year-old male with a history of hyperlipidemia presented to the emergency department with chest pain. Electrocardiographic abnormalities indicated an acute coronary syndrome. Urgent coronary angiography revealed nondominant right coronary artery (RCA) occlusion. During primary percutaneous coronary intervention (PCI), a 0.014-inch floppy guidewire could not be easily advanced into the middle RCA due to poor backup support from the guiding catheter and the patient's breathing. The pressure was monitored several times after reinserting the guiding catheter. Nevertheless, the guidewire was inadvertently inserted into the false lumen from the ostium, leading to subsequent dissection during contrast injection. Intravascular ultrasound (IVUS) imaging confirmed dissection from the ostium to the middle RCA and passage of the guidewire into the false lumen. An additional guidewire was successfully inserted into the true lumen of the RCA using real-time IVUS-guided wiring. We demonstrated successful bailout stenting for catheter-induced dissection of the nondominant small RCA. Our case highlights the risk of coronary artery dissection associated with guiding catheter use, especially in a nondominant small RCA, and the importance of optimal guiding catheter selection for primary PCI. The real-time IVUS-guided wiring technique can be applied to a single 6-Fr guiding catheter and is useful for quickly inserting a guidewire into the true lumen.

## 1. Introduction

Coronary artery dissection is a potentially life-threatening complication of percutaneous coronary intervention (PCI) [1, 2]. Inserting the guidewire into the true lumen is difficult when coronary artery dissection occurs, but it is necessary to improve vessel patency and patient outcomes.

## 2. Case Presentation

**Figure 1:**
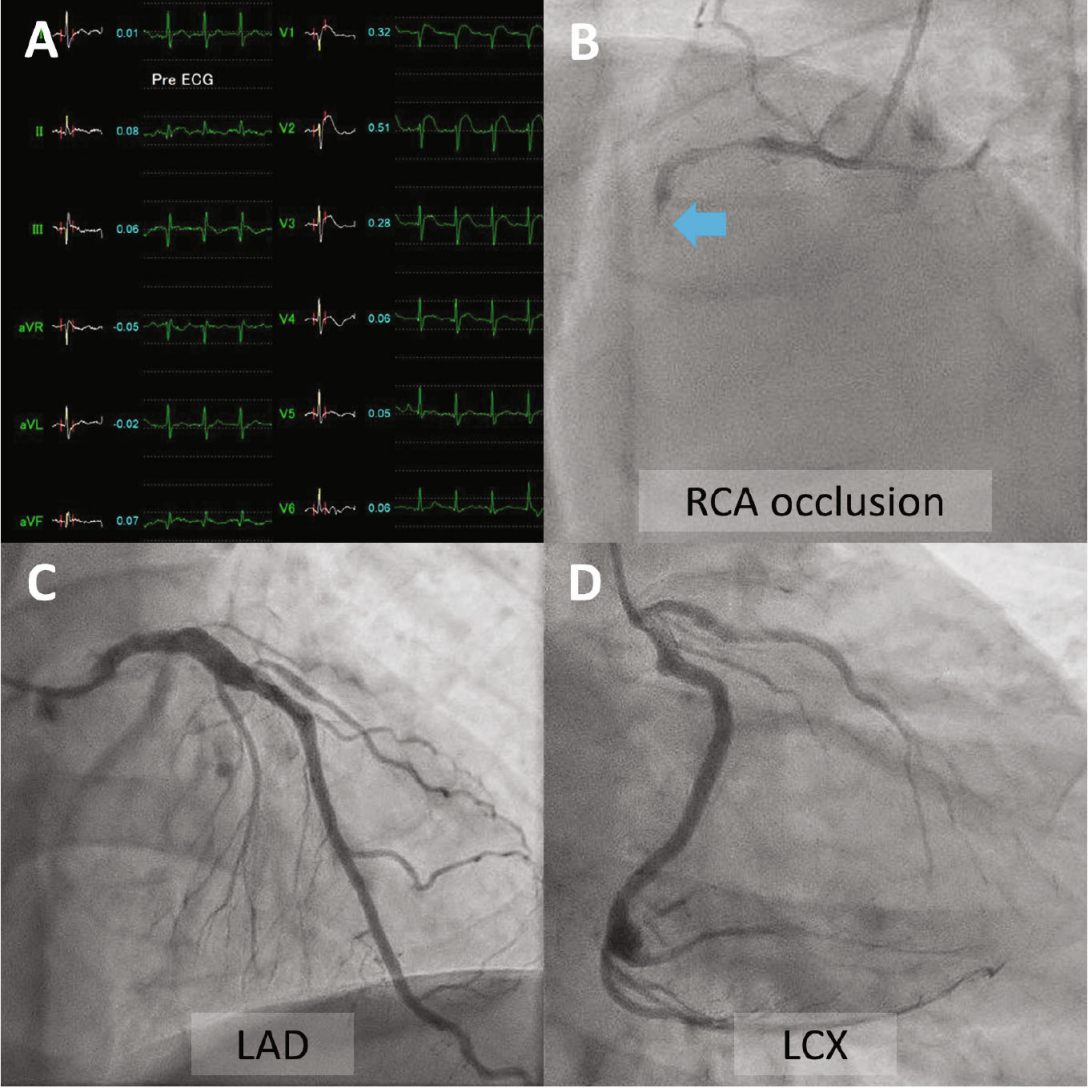
(A) Electrocardiography on admission shows ST-segment elevation in leads V1–V3 without reciprocal depressions in other leads, suggesting acute coronary syndrome involving a nondominant RCA. Coronary angiography shows occlusion of (B) the right coronary artery (RCA) (arrow), with no significant stenosis in (C) the left anterior descending artery (LAD) or (D) the dominant left circumflex artery (LCX).

**Figure 2:**
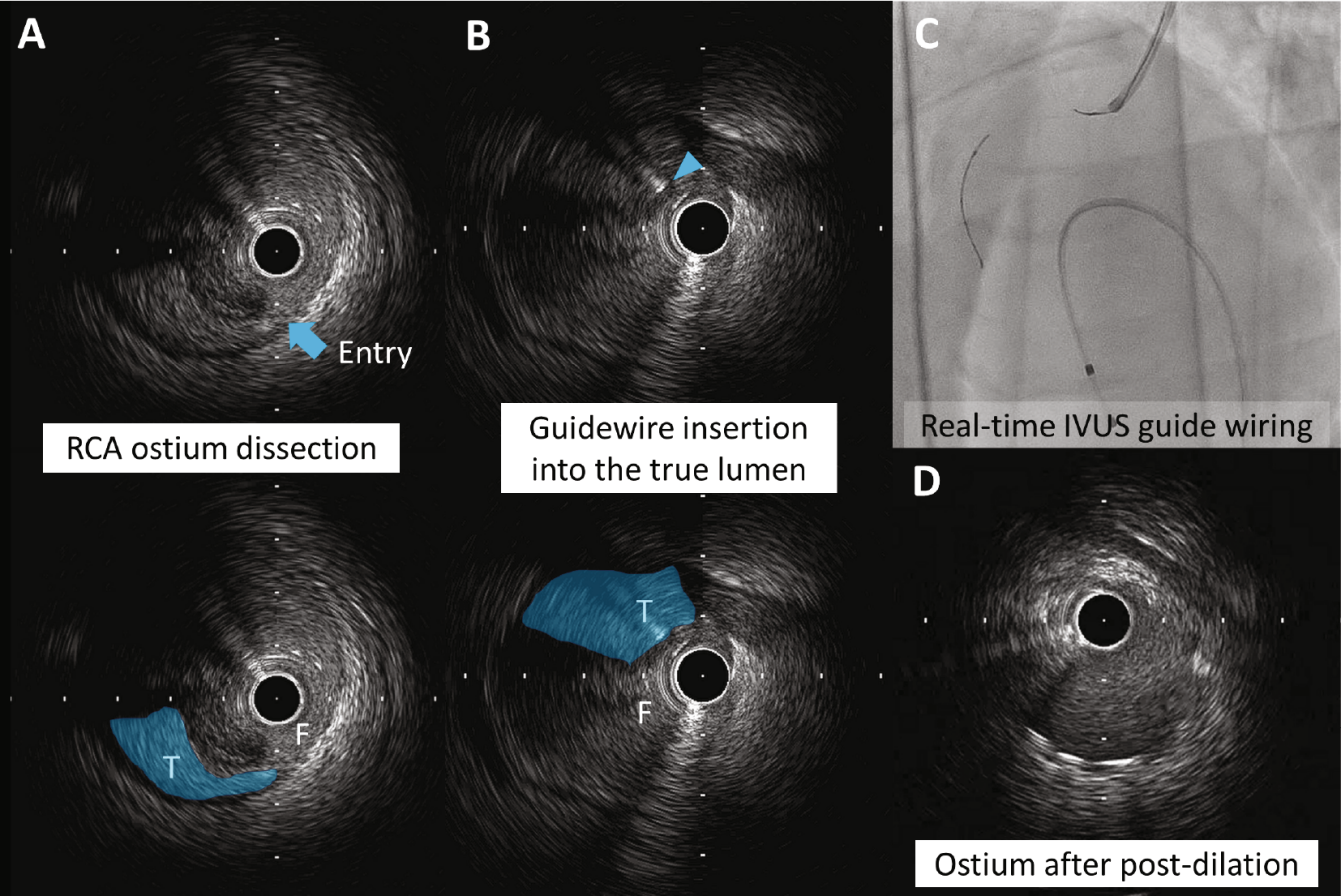
Bailout procedure for coronary artery dissection. (A) Intravascular ultrasound (IVUS) imaging shows the entry of dissection at the RCA ostium (arrow) and passage of the guidewire into the false lumen. The true lumen of the RCA is compressed by the enlarged false lumen. (B, C) An additional guidewire (arrowhead) is successfully inserted into the true lumen of the RCA using a real-time IVUS-guided wiring technique. (D) An IVUS image after postdilation of the proximal stent segment with a 3.0-mm noncompliant balloon. The blue-colored area indicates the true lumen (T). F, false lumen.

**Figure 3:**
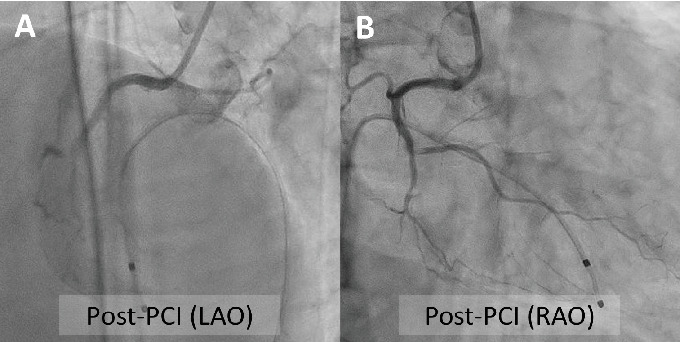
(A, B) Final angiography shows TIMI3 grade flow of the nondominant RCA. LAO, left anterior oblique angle; RAO, right anterior oblique angle.

**Figure 4:**
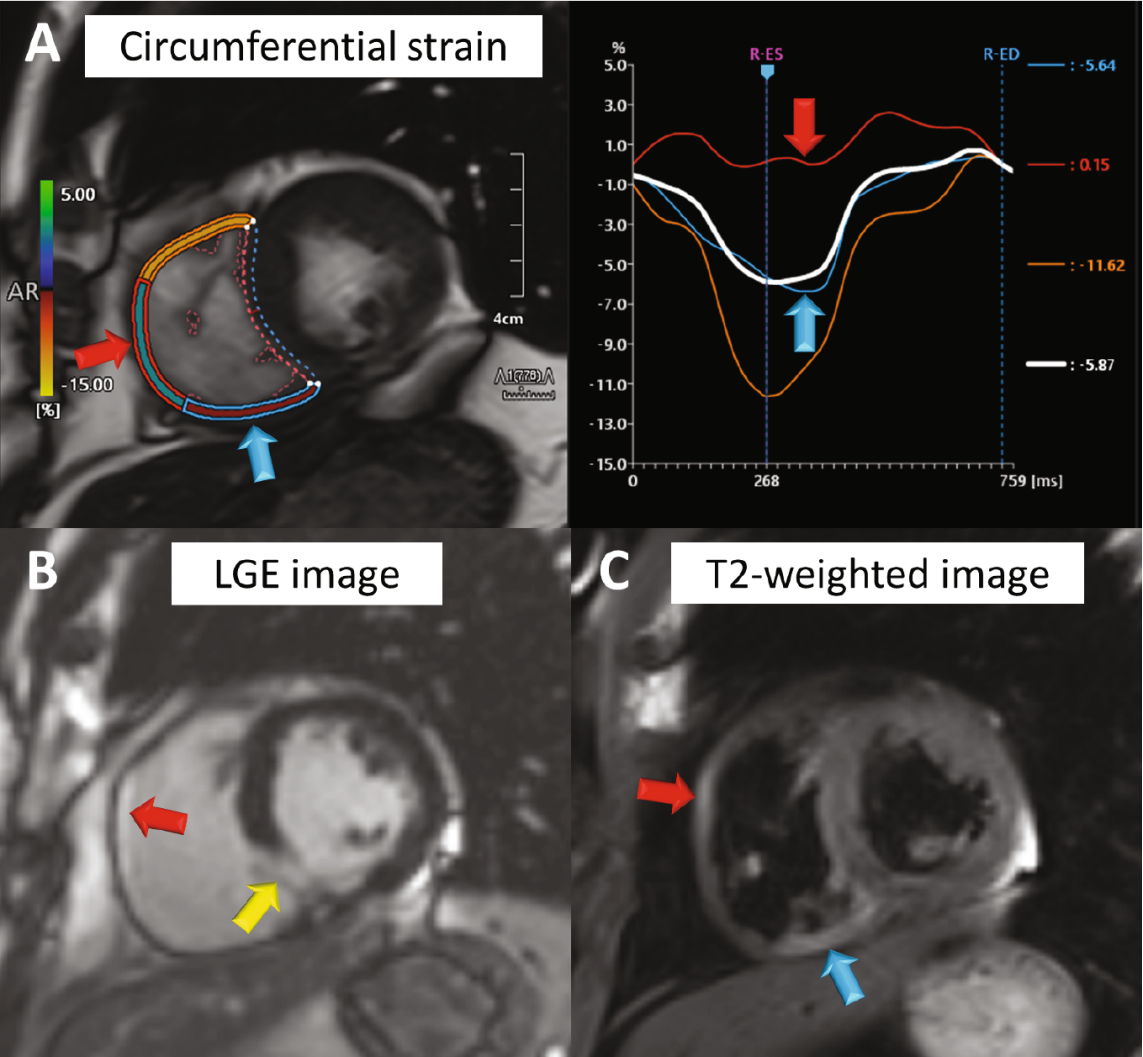
Cardiac magnetic resonance imaging 2 weeks after admission. (A) Reduced circumferential strain is seen in the inferolateral right ventricular free wall (arrows). (B) Late gadolinium enhancement imaging shows the infarcted right ventricular free wall (red arrow) and transmural infarction in the inferoseptal wall (yellow arrow). (C) T2-weighted sequence with fat suppression demonstrates a hyperintense signal of the right ventricular free wall (arrows), suggesting postinfarction inflammation/edema.

A 48-year-old man with a history of hyperlipidemia presented to the emergency department with chest pain. Electrocardiography showed ST-segment elevation in leads V1–V3 without reciprocal depression in the other leads (Figure 1(A)). Echocardiography revealed hypokinesis of the basal inferior wall. Urgent coronary angiography via the left radial artery using a 7-Fr Glidesheath slender (Terumo, Tokyo, Japan) demonstrated right coronary artery (RCA) occlusion (Figure 1(B), Supporting Information 1: Video S1), no significant stenosis in the left anterior descending artery (Figure 1(C), Supporting Information 2: Video S2), and a dominant left circumflex artery (Figure 1(D), Supporting Information 3: Video S3). The electrocardiographic abnormalities were consistent with acute coronary syndrome involving the nondominant RCA [3]. Given the notably reduced reflow area of the nondominant small RCA, our initial plan was to incorporate a stentless strategy with a drug-coated balloon. Since the target vessel was a small nondominant RCA, there was a concern that a ≤ 6-Fr guiding catheter or an Amplatz left guiding catheter might be too deeply seated into the proximal RCA. Therefore, a 7-Fr Judkins right guiding catheter with side holes was initially chosen to avoid wedged contrast injection. A 0.014-inch floppy guidewire (SION, Asahi Intecc, Seto, Japan) could not be easily advanced into the middle RCA because of poor backup support with the guiding catheter and the patient's breathing. The pressure was monitored several times after reinserting the guiding catheter. Nevertheless, the guidewire was inadvertently inserted into the false lumen from the ostium, leading to subsequent dissection during contrast injection (Supporting Information 4: Video S4). Intravascular ultrasound (IVUS) imaging using AltaView (Terumo) confirmed dissection from the ostium to the middle RCA and passage of the guidewire into the false lumen (Figure 2(A), Supporting Information 5: Video S5). The true lumen of the entire RCA was compressed by the enlarged false lumen. We exchanged the 7-Fr guiding catheter for a 6-Fr Judkins right guiding catheter with side holes to overcome the guiding catheter instability. This downsizing approach provided better maneuverability and backup support for guiding the catheter by maintaining a stable and coaxial position of the catheter tip against the RCA ostium. However, the 1st guidewire was also advanced into the false lumen of the RCA after the guiding catheter exchange. The IVUS probe via the 1st guidewire was set at the proximal RCA. The 2nd floppy guidewire was gently advanced to the opposite side of the first guidewire with clockwise rotation and successfully inserted into the true lumen of the RCA under real-time IVUS guidance (Figures 2(B) and 2(C), Supporting Information 6: Video S6). Given that the average diameter of the RCA at the bifurcation of the right ventricular branch was approximately 2.5 mm (Supporting Information 7: Video S7), a 2.5 × 38 mm Synergy drug-eluting stent (Boston Scientific, Natick, MA, United States) was deployed from the ostium to the right ventricular branch. Poststenting IVUS showed malapposition of the stent at the ostium (Supporting Information 8: Video S8). Postdilation of the proximal stent segment with a 3.0-mm noncompliant balloon resulted in acceptable IVUS (Figure 2(D), Supporting Information 9: Video S9) and angiographic results (Figure 3, Supporting Information 10: Video S10). The patient's symptoms and ST-segment elevation resolved after stenting. The door-to-balloon time was 84 min, and the peak creatine phosphokinase (CK) and creatine kinase–myocardial band (CK-MB) levels were 1514 U/L and 205 ng/mL, respectively. His left and right ventricular ejection fractions (as assessed by cardiac magnetic resonance imaging) 2 weeks after admission were 44% and 24%, respectively. Reduced circumferential strain was seen in the inferolateral right ventricular free wall (Figure 4(A), arrows). Late gadolinium enhancement imaging showed the infarcted right ventricular free wall (Figure 4(B), red arrow) and transmural infarction in the inferoseptal wall (Figure 4(B), yellow arrow). T2-weighted sequence with fat suppression demonstrated a hyperintense signal of the right ventricular free wall (Figure 4(C), arrows), suggesting postinfarction inflammation/edema. Fortunately, the patient was discharged without any right ventricular heart failure.

Catheter-induced coronary artery dissection occurs in approximately 1% of PCI cases [2] and is frequently associated with the deep engagement of a large catheter into a diseased small artery [1]. In this case, the initial inner diameter of the RCA ostium, as assessed by IVUS, was 2.2 mm, which was smaller than the outer diameter of the 7-Fr guiding catheter (2.4 mm). We used a real-time IVUS-guided wiring technique to insert an additional guidewire into the true lumen. Although IVUS-guided wiring usually requires a 7-Fr or 8-Fr guide system, the concomitant use of IVUS and another guidewire is compatible with 5-Fr and 6-Fr guiding catheters [4]. A small guiding catheter size may limit treatment options for complex lesions [4]. The larger guiding catheter has a more rigid catheter tip, which is a potential risk for coronary artery dissection. In this case, downsizing the guiding catheter provided better backup support, stability, and maneuverability in catheterization in comparison to a larger catheter. Another potential solution to avoid deep engagement of the guiding catheter is the insertion of an accessory free-floating guidewire from the guide catheter into the aortic root (the WALPO technique) [5]. To our knowledge, this is the first case reporting the usefulness of real-time IVUS-guided wiring with a single 6-Fr guiding catheter for iatrogenic dissection of a nondominant small RCA.

## 3. Conclusion

We demonstrated successful bailout stenting for catheter-induced dissection of a nondominant small RCA. Our case highlights the risk of coronary artery dissection associated with guiding catheter use, especially in a nondominant small RCA, and the importance of optimal guiding catheter selection for primary PCI. The real-time IVUS-guided wiring technique can be applied to a single 6-Fr guiding catheter and is helpful in quickly inserting a guidewire into the true lumen.

## Data Availability

The data supporting this study's findings are available on request from the corresponding author. The data are not publicly available due to privacy or ethical restrictions.

## Supporting Information

Supporting Information 1 Video S1. Initial coronary angiography of the right coronary artery (RCA). Coronary angiography demonstrated the RCA occlusion.

Supporting Information 2 Video S2. Initial coronary angiography of the left anterior descending artery (LAD). No significant stenosis was found in the LAD.

Supporting Information 3 Video S3. Initial coronary angiography of the left circumflex artery (LCX). No significant stenosis was found in the dominant LCX.

Supporting Information 4 Video S4. Diagnosis of iatrogenic coronary dissection. The guidewire was inadvertently inserted into the false lumen from the ostium, leading to subsequent dissection during contrast injection.

Supporting Information 5 Video S5. Intravascular ultrasound (IVUS) imaging through the guidewire in the false lumen. IVUS imaging confirmed dissection from the ostium to the middle of the RCA and passage of the guidewire into the false lumen.

Supporting Information 6 Video S6. Real-time IVUS-guided wiring. The 2nd floppy guidewire was gently advanced to the opposite side of the first guidewire with clockwise rotation and successfully inserted into the true lumen of the RCA under real-time IVUS guidance.

Supporting Information 7 Video S7. IVUS imaging performed through the first guidewire in the false lumen after the second guidewire insertion into the true lumen. The average diameter of the RCA at the bifurcation of the right ventricular branch was approximately 2.5 mm.

Supporting Information 8 Video S8. IVUS imaging after stenting from the RCA ostium to the right ventricular branch. Poststenting IVUS showed malapposition of the stent at the ostium.

Supporting Information 9 Video S9. IVUS imaging after postdilation of the proximal stent segment with a 3.0-mm noncompliant balloon. Postdilation of the proximal stent segment with a 3.0-mm noncompliant balloon resulted in acceptable IVUS.

Supporting Information 10 Video S10. Final coronary angiography of the right coronary artery. Postdilation of the proximal stent segment with a 3.0 mm noncompliant balloon resulted in acceptable angiographic results.
